# 

**Published:** 1875-07-01

**Authors:** 


*ORIGINAL COMMUNICATIONS:*

Obstruction of the Intestinal Canal by a Gall Stone. Reported by H. A. Johnson, M.D... 357
Phlebitis following the Hypodermic Use of Ergot in the Treatment of a Fibroid Tumor of the Uterus. By E. P. Allen, M.D. ...... 361
Treatment of Fibrous Tumors of the Uterus by Ergot. By W. H. Byford, M.D............. 365

*TRANSLATIONS:*

Gleanings from the German. By H. Gradle, M.D. ................................ 369

*EDITORIAL DEPARTMENT:*

American Medical Association ............... 370
Medical Department of the University of Chicago; University of Michigan.......... 371

*CORRESPONDENCE:*

A Letter from Paris. By C. C. Matteson, M.D. 372

*SOCIETY REPORTS:*

Chicago Society of Physicians and Surgeons. Regular Meeting, June 14, 1875. Reported by E. Warren Sawyer, M.D................ 377

*GLEANINGS FROM OUR EXCHANGES:*

Biometry. By Moreau Morris, M.D. ......... 378
Quinia as a Stimulant to the Pregnant Uterus. By Albert H. Smith, M.D................ 382
Retroversion of the Gravid Uterus—Retention of Urine—Recovery. By Dr. Arthur W. Edis, 385

